# A terahertz-vibration to terahertz-radiation converter based on gold nanoobjects: a feasibility study

**DOI:** 10.3762/bjnano.7.90

**Published:** 2016-07-06

**Authors:** Kamil Moldosanov, Andrei Postnikov

**Affiliations:** 1 Kyrgyz-Russian Slavic University, 44 Kiyevskaya St., Bishkek 720000, Kyrgyzstan; 2 Université de Lorraine, Institut Jean Barriol, LCP-A2MC, 1 Bd Arago, F-57078 Metz, France

**Keywords:** longitudinal acoustic phonon, microwave photon, nanobar, nanoring, terahertz emitter

## Abstract

**Background:** The need for practical and adaptable terahertz sources is apparent in the areas of application such as early cancer diagnostics, nondestructive inspection of pharmaceutical tablets, visualization of concealed objects. We outline the operation principle and suggest the design of a simple appliance for generating terahertz radiation by a system of nanoobjects – gold nanobars (GNBs) or nanorings (GNRs) – irradiated by microwaves.

**Results:** Our estimations confirm a feasibility of the idea that GNBs and GNRs irradiated by microwaves could become terahertz emitters with photon energies within the full width at half maximum of the longitudinal acoustic phononic DOS of gold (ca. 16–19 meV, i.e., 3.9–4.6 THz). A scheme of the terahertz radiation source is suggested based on the domestic microwave oven irradiating a substrate with multiple deposited GNBs or GNRs.

**Conclusion:** The size of a nanoobject for optimal conversion is estimated to be approx. 3 nm (thickness) by approx. 100 nm (length of GNB, or along the GNR). This detailed prediction is open to experimental verification. An impact is expected onto further studies of interplay between atomic vibrations and electromagnetic waves in nanoobjects.

## Introduction

The terahertz (THz) range of the electromagnetic waves, the range between microwaves and infrared (IR), is often discussed in reference to the “terahertz gap”, where “electronics meets optics”. The corresponding radiation, exhibiting properties common to one or the other of its neighbouring ranges, can be refracted and focused by lenses, like IR rays, and penetrate many optically opaque barriers, like microwaves. However, the methods of radiation generation and detection, elaborated for these adjacent ranges, are not efficient in the THz range and do face grave challenges. This hinders the creation of devices that can be a priori expected to open new fields of application, notably in medicine (non-invasive early diagnosis of cancer) and security (detection of concealed goods). The development of novel THz generation and detection methods, not borrowed from bordering technologies, invariably attracts the attention of the THz community.

Alternating electric fields of the THz frequency range are well known to exist in solids: such are the fields created by longitudinal acoustic vibrational modes (LAVMs). How can one “extract” them from a solid and transform into radiative energy of the THz range? It turns out that in metal nanoparticles, the LAVMs can be converted into electromagnetic waves with the help of Fermi electrons. Our idea consists of the simultaneous excitation of these latter by exposing them to a THz field of longitudinal phonon and microwave radiation. This should occur in a nanoparticle, where the conditions exist for an excited Fermi electron to relax fast enough via emitting a THz quantum. Specifically, gold nanobars (GNBs) and gold nanorings (GNRs) seem to be good candidates for implementing this idea. In the following, we discuss the theoretical prerequisites for the effect within the quasiparticle approach.

## Results and Discussion

**Figure 1:**
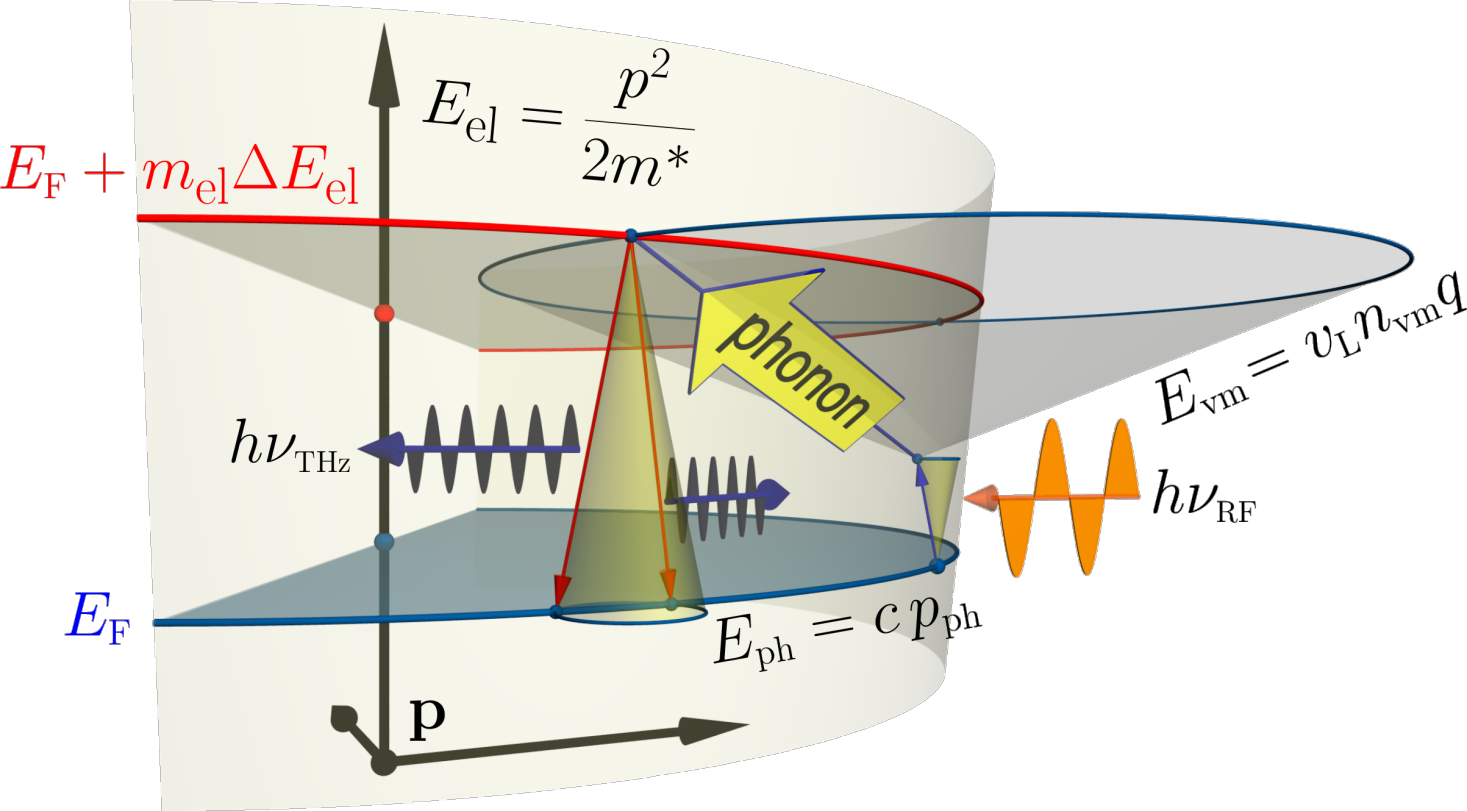
Scheme of absorption of microwave radiation *h*ν_RF_ by an electron at *E*_F_, assisted by an absorption of a longitudinal phonon, with subsequent emission of a terahertz quantum *h*ν_THz_. The corresponding dispersion laws are indicated. See text for details.

The microwave photons are considered to be quasiparticles with the dispersion law *E*_ph_ = *c*·*p*_ph_ (*c* is the speed of light); the longitudinal phonons would be quasiparticles with the dispersion law *E*_vm_ = *v*_L_·*n*_vm_·*q*, where *v*_L_ is the (longitudinal) velocity of sound in gold; finally, the electrons would be quasiparticles with the dispersion law *E* = *p*^2^/(2m^*^), m^*^ being the electron effective mass. These three quasiparticle species may interact, subject to their specific quantization conditions and the energy/momentum conservation laws. The latter are grasped by the scheme in Figure 1 where an excitation of a Fermi electron “upwards” through *m*_el_ quantization steps Δ*E*_el_ is brought about by a joint absorption of a RF quantum and an appropriate longitudinal phonon. Subsequently the excited electron relaxes to the Fermi energy, releasing a quantum at THz frequency. In the following, we argue about conditions under which the THz-emitting channel of relaxation may precede other imaginable mechanisms, e.g., that of releasing the extra energy into the phonon bath again. This case we considered in a related but different context [1]. It is essential for our analysis that the nanoobjects are much larger in one dimension than in the other two, hence having the shape of nanobars or closed thin nanorings.

**Figure 2:**
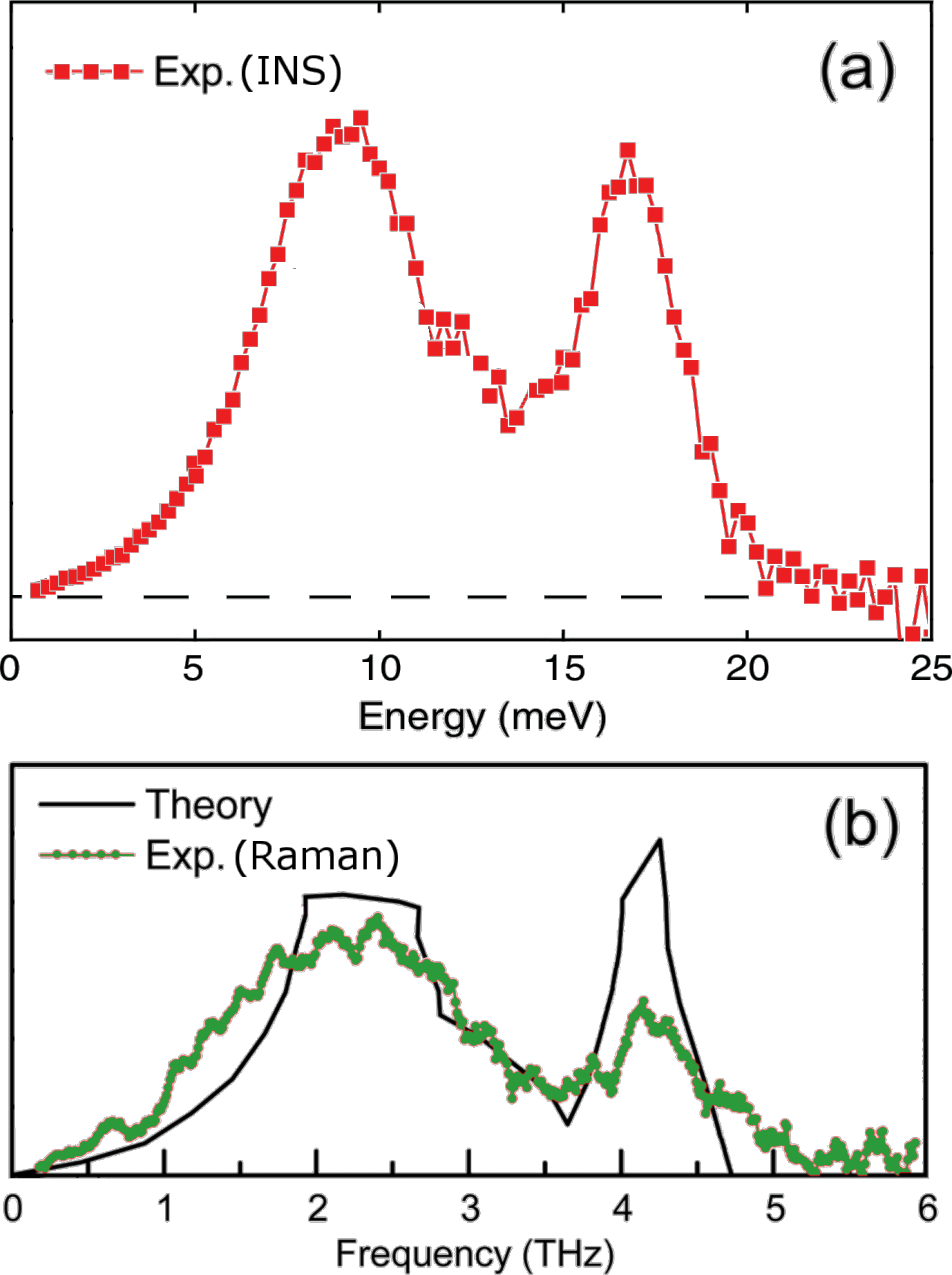
Vibrational density of states of gold as extracted from (a) inelastic neutron scattering on thick foils [2], (b) plasmon resonance Raman scattering on nanocrystals [3] (green dots) and reconstructed from force constants fitted to inelastic neutron scattering data from massive single crystals [4] (continuous black line). Adapted from Figure 2 of Muñoz et al. [2] and Figure 5a of Bayle and co-workers [3].

We start with some numerical estimates. The strongest “assistance” of longitudinal phonons to electron excitations is expected within the full width at half maximum of a pronounced peak in the density of longitudinal acoustic modes of gold (energies of ca. 16–19 meV, i.e., frequencies of ca. 3.9–4.6 THz) – see Figure 2, based on the results by Muñoz et al. [2] on inelastic neutron scattering and by Bayle et al. [3] on plasmon resonance Raman scattering.

For the microwave irradiation, a standard domestic microwave oven would offer a simple practical source at ν_RF_ = 2.45 GHz, i.e., *h*ν_RF_ ≈ 1.01·10^−2^ meV. As this is much smaller than the above phonon-related values, the peak outcome of the THz radiation is not expected to be shifted from the peak of the phonon density of modes, i.e., around 4.2 THz (see below). The sizes of GNBs and GNRs have to be much smaller than the skin depth in gold at 2.45 GHz, amounting to approx. 1.5 μm. This justifies considering the Fermi electrons in the following as free ones.

**Figure 3:**
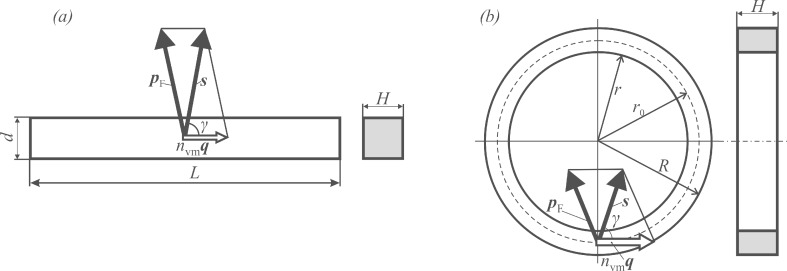
Geometry of nanoobjects and phonon/electron momenta in GNB (a) and in GNR (b). For simplicity, the momentum **p**_ph_ of the microwave photon is omitted, by force of relations 
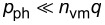
, 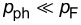. Note that the compression wave may travel in both directions along the bar or the ring, whereas the propagation of excited electrons is confined along the conical surfaces with large opening angle, 2γ ≈ 140°.

Figure 3 sets the geometry of GNBs (left panel) and GNRs (right panel), for the discussion that follows. In a GNB, the compression waves (longitudinal phonons with the momentum *n*_vm_**q** and the energy *E*_vm_ = *v*_L_·*n*_vm_·*q*) propagate along its length. It will be argued below that the momentum of an excited electron stands at a large angle γ to that of the longitudinal phonon. However, the electron cannot leave the GNB because of substantial work function of gold (approx. 4.3 eV) [5]. By creating conditions to prevent the scattering of the excited electron at the GNB boundaries and its relaxation via emission of a longitudinal phonon, the setup will be given for channeling the relaxation process into an emission of a THz photon.

In GNR (Figure 3b), the compression waves run along the cyclic contour, to which the phonon momentum *n*_vm_**q** is tangential. Similarly to the case of GNB, the momentum of an excited electron stands at large angle γ to the momentum of the phonon.

The absorption condition for the microwave-range photon is the following:

[1]
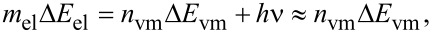


where *m*_el_ is the number of electron energy “gaps” (quantization steps between confinement-induced discrete energy levels in the nanoobject), and *n*_vm_ is a number of vibrational quanta (for LAVMs). According to the Kubo formula [6–7] (see also the Appendix in [1]), the step in the electron energy levels Δ*E*_el_ relates to the number of gold atoms *N*_a_ as follows: Δ*E*_el_ ≈ (4/3)·(*E*_F_/*N*_a_). Then the condition (Equation 1) that *m*_el_ energy steps must embrace *n*_vm_ vibrational quanta takes the form:


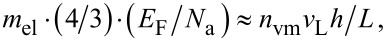


where *L* is the confinement length delimiting the propagation of compression waves (i.e., the maximal dimension of a GNB or the median circumference of a GNR, correspondingly), and the sound velocity *v*_L_ relates frequency to wave vector. Further on, assuming that the density in a GNB equals that of bulk gold, the number of atoms in GNB can be expressed via density of gold ρ, atomic mass *m*_a_ and the volume of the nanoobject *V*. The resulting *m*_el_/*n*_vm_ ratio for the two cases is as follows:


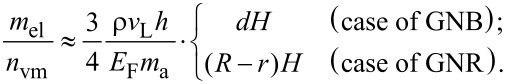


The numerical values for gold *E*_F_ = 5.53 eV [5], *m*_a_ = 197 in atomic units, or approx. 3.27·10^−22^ g, ρ = 19.3 g/cm^3^, *v*_L_ = 3.23·10^5^ cm/s [8] yield, for both *dH* (case GNB) and (*R* − *r*)*H* (case GNR) a value of approx. 9.35·(*m*_el_/*n*_vm_).

To provide a high intensity of the THz emission, the number of quantized energies within the FWHM of the longitudinal phononic density of modes ought to be sufficiently large. In order to increase the efficiency of conversion, each “allowed” mode within the FWHM has to be used, that imposes (*m*_el_/*n*_vm_) to be an integer. On the other hand, an average excited electron should not be scattered on its way towards the GNB boundary. Hence, *d*/2 and *H*/2 should reasonably not exceed the electron mean free path *l*_0_ in gold nanoparticles. Rough estimates in our earlier work yield *l*_0_ ≈ 1.7 nm [1], which reveals the “working” matching relation (*m*_el_/*n*_vm_) = 1 and not larger. Specifically, accepting this relation and *H* ≈ 3.1 nm, we get

[2]
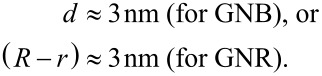


**Figure 4:**
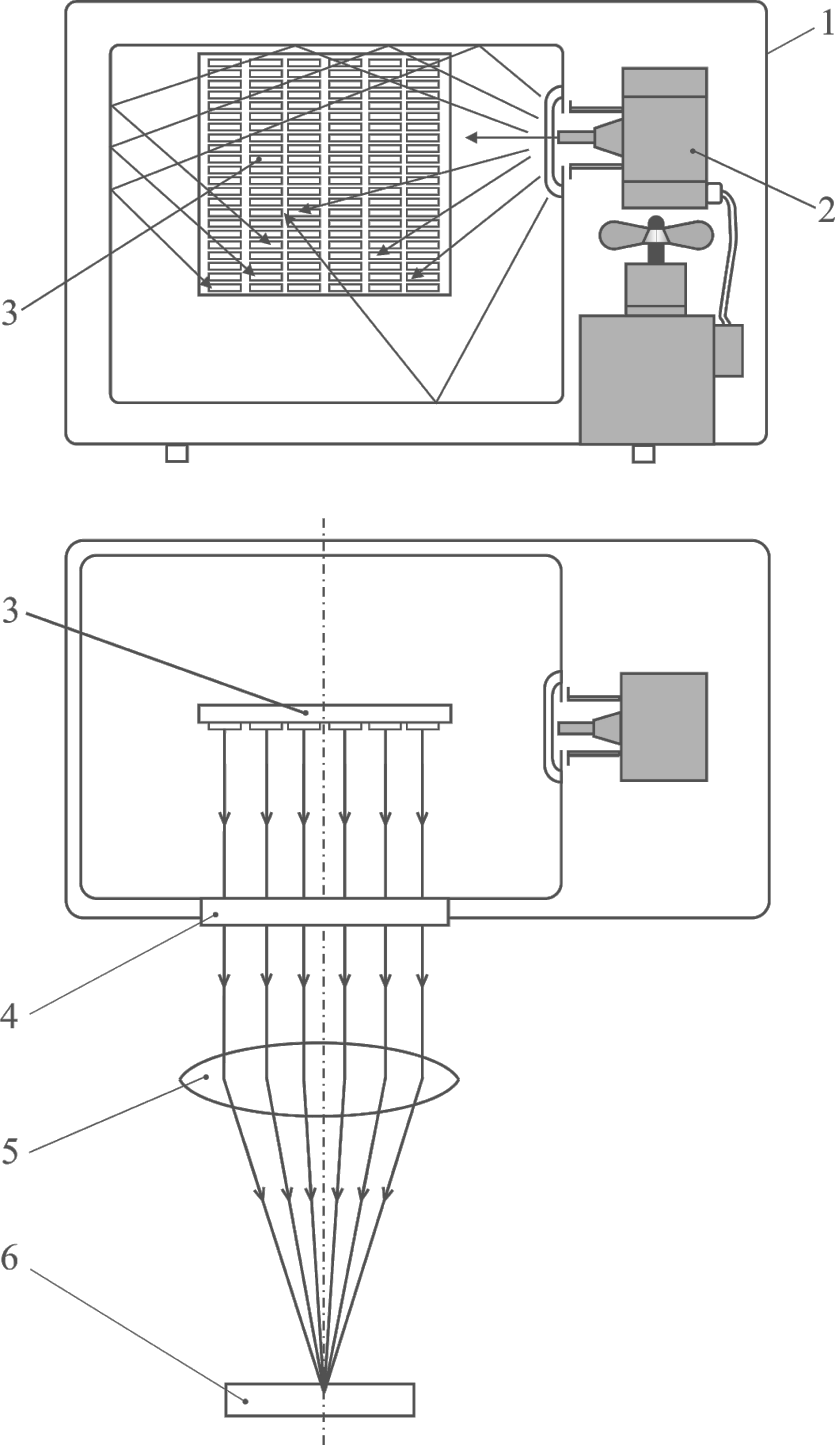
An outline of the THz radiation source based on a domestic microwave oven. 1: microwave oven, 2: magnetron (ν = 2.45 GHz), 3: substrate with densely deposited GNBs or GNRs, 4: THz band resonance filter (ν_0_ = 4.2 THz), 5: lens, 6: object to be studied.

Each “allowed/working” phonon can promote the Fermi electron onto the vacant level corresponding to the energy of this phonon, measured from *E*_F_. On relaxation, the energy of the emitted THz photon would roughly match (neglecting a much smaller energy of the microwave photon) that of the LAVM. Consequently, the “primary” emitted THz spectrum (intensity distribution over energies) is expected to roughly follow the distribution of the LAVM density in gold. However, the resulting spectrum issued by the device would be limited by the transmission width of the band resonance filter (see below in Figure 4, pos. 4).

In addition to the energy conservation law for GNBs/GNRs expressed by Equation 1, we take now into account the momentum conservation, making reference to the schemes depicted in Figure 3. The LAVM with momentum *n*_vm_**q** propagates along the length of the GNB (or, in GNR, over the median cycle contour along the ring). Everywhere within the GNB/GNR, a Fermi electron with momentum **p**_F_ can be encountered, prone to absorb a microwave photon. The contribution of the latter *p*_ph_ to the electron momentum will be neglected here due to its relative smallness (as was done above for the energy *hν*). Assuming the vector **s** to be the sum of **p**_F_ and *n*_vm_**q**, the following relation holds: 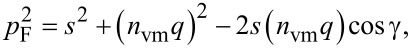 whence the angle that defines the propagation direction of the excited electron reads:


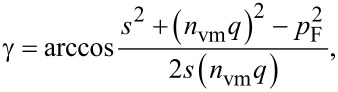


where 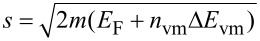. By expressing momentum and energy quanta of the the vibration mode, *q* and Δ*E*_vm_, respectively, in terms of the length of path along which the compression waves propagate, *L*, and the longitudinal velocity of sound *v*_L_, 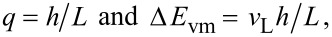 one arrives at the estimate for γ in terms of *n*_vm_ and *L*:


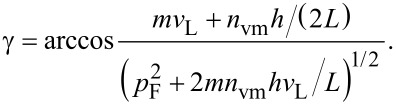


Note that in GNB, *L* is the length of the nanobar, whereas in GNR, the median contour length π(*R* + *r*) should be taken instead (see Figure 3b), hence


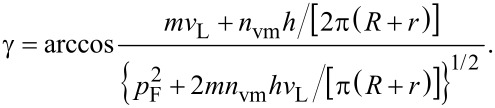


Taking into account the condition *n*_vm_Δ*E*_vm_ + *hν* ≈ 17.4 meV along with the numerical values of gold parameters and constants this yields for both GNB and GNR γ ≈ arccos(0.342) ≈ 70°.

The excited electron transverses the GNB/GNR at this angle to the line of phonon propagation and reaches the boundary. The ensuing imaginable scenarios are: (i) elastic back-scattering into the GNB/GNR volume, that would simply “postpone” the electron’s relaxation; (ii) releasing the “originally borrowed” longitudinal phonon and the microwave photon, restoring the initial situation; (iii) inducing just a longitudinal phonon of slightly higher energy, ultimately translating the initially absorbed radiation into heat; (iv) releasing the surplus energy into a transversal surface phonon; (v) emitting the surplus energy in form of a THz photon. We emphasize that the electron with the given excitation energy will not exit the sample because the work function of gold is too high to overcome. Let us briefly discuss these possibilities. The cases (ii) and (iii) will be unfavored (and hence rare) by force of geometry considerations, in view of the nearly normal orientation of the momentum of the electron to the surface. Although the case (iii) would mean an “useless” (in the sense of our objective) waste of the microwave energy, it will “populate” the system with longitudinal phonons, thus enhancing the possibility of scenario (v) and compensating for the “cooling” implied by the latter. Note that this very process came about as the useful one in a different context, in our work on hyperthermia with the help of gold nanoparticles [1]. The case (iv) might seem plausible (albeit similarly “useless”); the consideration against it is that a transversal phonon will likely be “out of resonance” with the energy delivered by a longitudinal phonon, since their dispersion relations are different, and the confinement-imposed energy quantization would likely prevent the necessary energy match of their respective *n*_vm_Δ*E*_vm_ values. Putting it differently, the major peaks in the densities of modes of transversal and longitudinal phonons in gold (and particularly in gold nanoparticles) are well separated (at ca. 2.4 and ca. 4.2 THz, respectively), as it was discussed by Bayle et al. [3] (see Figure 2). Credible numerical simulations comply quite well, even quantitatively, with such a neat separation of the vibration spectrum of gold into two peaks, in what concerns nanoparticles [9] as well as the bulk [10]. Coming back to the revision of our scenarios and facing the drawbacks in all so far discussed, we are left with the scenario (v), the only “useful” one, according to which the electron relaxes back to the Fermi level via a radiative transition emitting a photon at approx. 4.2 THz, i.e., with the energy of approx. 17.4 meV (or close to it, according to that of the “primary” photon).

In order to reduce the probability of elastic scattering of the electron at the GNB boundary, and hence to enforce the decay probability into THz photon emission, one can try to enhance the electron density of states at the Fermi level. This can be achieved, e.g., by ion implantation of Ta or Fe impurities into the subsurface layer of nanoobjects. The idea is that the doping of bulk gold with Ta or Fe atoms creates impurity d-levels (in one of the spin channels, since the impurities are magnetic) at the Fermi energy [11]. Should the scheme work, the GNB/GNR array would turn into a steady source of a THz-range quasicontinuous spectrum centered at 4.2 THz, or 17.4 meV (the major peak in the LAVM of gold) and possessing the FWHM of the latter (approx. 0.7 THz, or 2.9 meV). “Quasicontinuous” (in the frequency domain) implies here that the emitted THz spectrum is expected to roughly follow the phonon density of modes. However, in consistency with the confinement conditions imposed on the nanoobject, i.e., the discreteness of the frequency of the order of *v*_L_/*L* ≈ 10^10^ Hz (see the discussion about the practical values of *L* below). “Steady” means that the radiation is emitted continuously in the time domain, as long as the microwave oven operates, and not in the pulsed regime.

The above estimates of the size of the nanoobjects (Equation 2) concern the “thickness” (*d*)/(*R* − *r*)/(*H*) but neither the length of the GNB, *L*, nor the mean radius of the GNR, *r*_0_ = (*R* + *r*)/2. However, these dimensions come into the foreground as we think over how to reduce the heating of nanoparticles by 2.45 GHz radiation, that would occur due to uncertainty in the momentum value of the Fermi electrons. For sizes as given in Equation 2 the uncertainty in the Fermi electron momentum guarantees that the momentum conservation will hold. In order to prevent the “direct” absorption of the microwave radiation by the nanoobjects, one has to keep the relevant energy intervals incompatible – e.g., by choosing the sizes of the nanoparticles such that the energy step Δ*E*_el_ between the corresponding energy levels in the electron system would be far larger than *hν* = 1.01·10^−5^ eV. A reference to the abovementioned Kubo formula [6–7] yields the “critical” (in this sense) length dimensions (in nm) of GNBs: *L <* 1.24·10^4^·*d*^−1^·*H*^−1^, or of GNRs: *r*_0_ *<* 1.97·10^3^·(*R* −*r*)^−1^·*H*^−1^. For example, for *d* ≈ 3 nm, *H* ≈ 3.1 nm from Equation 2, the maximal linear size *L*_max_ is approx. 1.33·10^3^ nm. By choosing *L* ≈ 100 nm, we can safely avoid a “direct” heating of GNBs by microwave radiation. In case of GNRs, the choice *R* − *r* ≈ 3 nm, *H* ≈ 3.1 nm yields the maximum mean radius (*r*_0_)_max_ ≈ 2.11·10^2^ nm. A choice of radius of say *r*_0_ = 20 nm, would securely help against a “direct” heating.

The suggested idea of transforming THz “sound” into THz electromagnetic waves can hopefully be realized as a source of continuous THz radiation. Figure 4 depicts a possible scheme. As a practical source of microwave radiation, a magnetron of a domestic microwave oven is suggested, that operates at ν = 2.45 GHz. In this case, *hν* ≈ 1.01·10^−2^ meV 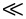 17.4 meV, so that the approximation 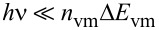 holds and the approximation in Equation 1 is valid. A substrate with multiple GNBs is placed into the oven chamber, where the GNBs are exposed to both direct irradiation from the magnetron and to that reflected from the chamber walls. Effectively, the microwave photons could be absorbed anyway inside the GNBs, where consequently the THz photons will be emitted. The resulting THz radiation is channeled out of the chamber via a THz band resonance filter with resonance frequency ν_0_ = 4.2 THz (*hν*_0_ = 17.4 meV), whereby the scattered microwave radiation is retained. The outcoming THz waves will be focused by a lens (fabricated, e.g., from high-density polyethylene).

Finally, two technical remarks are in place. The first one concerns the validity of a quasiparticle picture implied above for the description of electrons in nanoobjects: are the latter “large enough” for the discussion in terms of Figure 1 to make sense? The Fermi radius in (bulk) gold being *k*_F_ ≈ 1.20·10^10^ m^−1^, the de Broglie wavelength of an excited electron measures ca. 0.52 nm, which fits, on the average, roughly three times along the free path to the boundary of the nanoobject. Hence the situation is just at the border of the applicability region for the quasiparticle approximation; we use the latter out of practical convenience, keeping in mind the care to be taken.

The second remark is that the above estimated dimensions of GNBs and GNRs are on the edge in yet another way, namely, their size is at the resolution limit for available lithographic techniques (intrinsic resolution of the electron beam lithography [12] is 3–5 nm). Presumably, it is technologically simpler to fabricate the GNBs than to fabricate the GNRs. A number of strategies exists to produce gold nanorods and nanowires of ca. 3 nm diameter [13–15], which could be used as the GNBs. These strategies, possibly, could be tailored for making the GNBs on the substrate or embedded into a matrix transparent in the THz wavelength range. As for GNRs, Tseng et al. [16] fabricated nanorings using the colloidal lithography process. Hopefully, it could be tailored to provide the GNR sizes estimated here.

Summarizing, fully aware that extremely small size nanoobjects are difficult to fabricate, we offer brief assessments hinting that the conversion of the THz lattice vibrations to THz electromagnetic radiation could be possible via electron excitation/relaxation processes within the GNBs or GNRs. On the basis of the principles outlined, a practical source of THz radiation might be hopefully created, which would find applications, e.g., for the study of biological objects (cancer diagnostics) and/or for detection of concealed objects. The further studies of underlying physics may have impact on applications as well as on research of interactions of electrons with atomic vibrations and electromagnetic waves in nanoobjects.

### Further possible applications of phonon–photon coupling

Already on conclusion of this work, our attention was brought to some activities in “neighbouring” fields that might put the present study into a yet another context. In quite recent works [17–18], a tunneling of acoustic phonons across nanoscale gaps became an issue of priority. Meanwhile, already in 2010 Beardsley et al. [19] described a working 0.44 THz saser (on the basis of semiconductor superlattices). It seems that metallic nanobars/nanorings supposed to serve as resonators for LAVMs, taken in combination with a THz saser (possibly in a different realisation than that of [19]) and interconnected by the phonon tunneling “leaks” between the nanoobjects, may serve as elements of the THz acoustic phononic circuitry.

Another idea is that metallic nanobars/nanorings could be used for interfacing THz photons with THz phonons, that is crucial for realization of quantum networks. Thus, Riedinger et al. [20] demonstrated the quantum pairing of light with vibrations of microscopic mechanical oscillators, a potentially useful effect for the development of quantum-information processing systems. A working unit for quantum-information applications can be imagined in the spirit of a scheme described by Stannigel et al. [21–22], in which a nanomechanical resonator mediates interactions between the qubits and light. We suggest that gold nanobars and nanorings discussed in the present work may be plausible candidates for such applications as well.
